# Combined use of tyrosine kinase inhibitors with PD-(L)1 blockade increased the risk of thyroid dysfunction in PD-(L)1 blockade: a prospective study

**DOI:** 10.1007/s00262-024-03733-2

**Published:** 2024-06-04

**Authors:** Tomoko Kobayashi, Shintaro Iwama, Ayana Yamagami, Tetsushi Izuchi, Koji Suzuki, Koki Otake, Yoshinori Yasuda, Masahiko Ando, Takeshi Onoue, Takashi Miyata, Mariko Sugiyama, Daisuke Hagiwara, Hidetaka Suga, Ryoichi Banno, Tetsunari Hase, Naoki Nishio, Shoichiro Mori, Tomoya Shimokata, Tomoyasu Sano, Kaoru Niimi, Nobuhisa Yoshikawa, Shusuke Akamatsu, Yuichi Ando, Masashi Akiyama, Michihiko Sone, Makoto Ishii, Hiroshi Arima

**Affiliations:** 1 Department of Endocrinology and Diabetes, Nagoya University Graduate School of Medicine, 65 Tsurumai-Cho, Showa-Ku, Nagoya, 466-8550 Japan; 2 Center for Advanced Medicine and Clinical Research, Nagoya University Hospital, Nagoya, Japan; 3 Physical Fitness and Sports, Research Center of Health, Nagoya University, Nagoya, Japan; 4 Department of Respiratory Medicine, Nagoya University Graduate School of Medicine, Nagoya, Japan; 5 Department of Otorhinolaryngology, Nagoya University Graduate School of Medicine, Nagoya, Japan; 6 Department of Dermatology, Nagoya University Graduate School of Medicine, Nagoya, Japan; 7 Department of Clinical Oncology and Chemotherapy, Nagoya University Hospital, Nagoya, Japan; 8 Department of Urology, Nagoya University Graduate School of Medicine, Nagoya, Japan; 9 Department of Obstetrics and Gynecology, Nagoya University Graduate School of Medicine, Nagoya, Japan

**Keywords:** Thyroiditis, Hypothyroidism, irAE, PD-1, PD-L1, TKI

## Abstract

**Background:**

Anti-programmed cell death-1 (ligand-1) antibody [PD-(L)1-Ab] can cause destructive thyroiditis and/or hypothyroidism. In addition, tyrosine kinase inhibitors (TKIs) frequently induce hypothyroidism. The aim of this prospective study is to examine the incidence and clinical characteristics of thyroid dysfunction induced by combination therapy of a PD-(L)1-Ab and TKI [PD-(L)1-Ab/TKI].

**Methods:**

A total of 757 patients treated with PD-(L)1-Ab or PD-(L)1-Ab/TKI were evaluated for anti-thyroid antibodies (ATAs) at baseline and for thyroid function for 48 weeks after treatment initiation and then observed until the last visit.

**Results:**

The cumulative incidences of destructive thyroiditis [4/23 (17.4%) vs. 45/734 (6.1%) patients, *p* < 0.001], isolated hypothyroidism [10/23 (43.5%) vs. 29/734 (4.0%) patients, *p* < 0.001], and all thyroid dysfunction [14/23 (60.9%) vs. 74/734 (10.1%) patients, *p* < 0.001] were significantly higher in the PD-(L)1-Ab/TKI group than PD-(L)1-Ab group, respectively. All patients positive for ATAs at baseline developed thyroid dysfunction after PD-(L)1-Ab/TKI treatment, a significantly higher incidence than that in those negative for ATAs at baseline [4/4 (100%) vs. 10/19 (52.6%) patients, *p* = 0.026].

**Conclusions:**

The addition of TKIs increased the risk of thyroid dysfunction induced by PD-(L)1-Ab, with the risk being higher in patients positive for baseline ATAs.

**Supplementary Information:**

The online version contains supplementary material available at 10.1007/s00262-024-03733-2.

## Introduction

Immune checkpoint inhibitors (ICIs), which block the immune checkpoints allowing T cells to fight cancer cells, have been developed and used for treatment of advanced malignancies. On the other hand, ICIs can lead to adverse events, termed immune-related adverse events (irAEs), in the lung, skin, gastrointestinal tract, liver, and endocrine glands [1]. Among the endocrine irAEs, thyroid dysfunction is one of the most frequently observed [2–6].

Although thyroid dysfunction can be induced by any class of ICIs, they occur more often following anti-programmed cell death-1 antibody (PD-1-Ab) or anti-programmed cell death-1 ligand-1 antibody (PD-L1-Ab) therapy compared with cytotoxic T-lymphocyte antigen-4 antibody monotherapy [7, 8]. Previously, we reported that thyroid dysfunction was observed in 9.9% (41/416) of patients treated with PD-1-Ab [9] and 10.1% (15/148) of patients treated with PD-L1-Ab [10]. Thyroid dysfunction is classified as thyrotoxicosis and hypothyroidism, and most cases of thyrotoxicosis are destructive thyroiditis (DT). In our previous prospective studies, the incidences of DT and hypothyroidism were 6.7 and 3.1% during PD-1-Ab therapy [9] and 5.4 and 4.7% during PD-L1-Ab therapy [10], respectively.

Tyrosine kinase inhibitors (TKIs), which target vascular endothelial growth factor (VEGF) receptors 1–3, fibroblast growth factor receptors 1–4, platelet-derived growth factor receptor α, the transmembrane tyrosine kinase rearranged during transfection, and a region of stem cell factor receptor [11, 12], are also reported to frequently induce thyroid dysfunction, mainly hypothyroidism [13]. The incidence of TKI-induced hypothyroidism varies among TKIs: 16.4–52.0% for lenvatinib [14–16], 14.6–23.0% for cabozantinib [17, 18], and 19.2–48.4% for axitinib [19–21], respectively. On the other hand, the incidence of DT in TKI treatments has been rarely reported in previous clinical trials.

Recently, PD-1-Abs or PD-L1-Abs [PD-(L)1-Abs] have been used in combination therapies with TKIs for several types of cancer. Combination therapy of a PD-(L)1-Ab plus a TKI [PD-(L)1-Ab/TKI] increased the frequency of thyroid dysfunction compared with TKI monotherapy in several clinical trials. In a phase 3 trial, hypothyroidism and thyrotoxicosis were observed in 47.2% (166/352) and 8.0% (28/352) of patients with advanced renal cell carcinoma (RCC) treated with lenvatinib plus pembrolizumab, respectively [22]. In another phase 3 trial, hypothyroidism and thyrotoxicosis were observed in 34.1% (109/320) and 10.0% (32/320) of patients with advanced RCC treated with cabozantinib plus nivolumab, respectively [23]. However, no real-world prospective studies have measured thyroid hormones at regular intervals to determine the incidence of thyroid dysfunction, as a primary outcome, after PD-(L)1-Ab/TKI compared with PD-(L)1-Ab treatment. It is also unknown whether the presence of anti-thyroid antibodies (ATAs) [anti-thyroglobulin antibody (TgAb) and/or anti-thyroid peroxidase antibody (TPOAb)] at baseline, which is a risk factor for thyroid dysfunction induced by PD-1-Ab [9, 10, 24, 25], could affect thyroid dysfunction induced by PD-(L)1-Ab/TKI as well.

The aim of the present study was to clarify the incidence of thyroid dysfunction induced by PD-(L)1-Ab/TKI treatment in patients positive or negative for ATAs at baseline in a prospective study.

## Materials and methods

### Patients

**Fig. 1:**
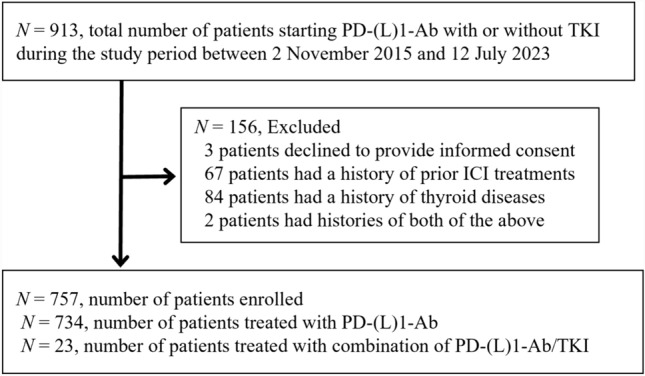
Enrollment of the study subjects. PD-(L)1-Ab, anti-programmed cell death-1 (ligand-1) antibody; TKI, tyrosine kinase inhibitor; ICI, immune checkpoint inhibitor

Since November 2, 2015, we conducted a prospective study analyzing irAEs in patients treated with ICIs to identify the clinical features of endocrine irAEs (UMIN000019024). The schema of this study is shown in Fig. 1. All patients who started nivolumab, pembrolizumab, or avelumab treatment between November 2, 2015 and July 12, 2023 at Nagoya University Hospital were included in this study since the above PD-(L)1-Abs can be used as combination therapy with TKIs. The TKIs were administered concurrently with PD-(L)1-Abs as combination therapy. Patients treated with PD-(L)1-Ab in combination with chemotherapeutic drugs other than TKIs (e.g., carboplatin, pemetrexed, cisplatin, 5-fluorouracil) were included in the PD-(L)1-Ab group since these cytotoxic chemotherapeutic agents rarely affect thyroid function. The patients were observed until the time of death, referral to another hospital, or a change in treatment from the initial PD-(L)1-Ab therapy. Patients who did not provide informed consent (*n* = 3) at enrollment, those with a history of ICI treatment (*n* = 67), those with a history of thyroid disease (*n* = 84) including Graves’ disease, hypothyroidism with levothyroxine replacement therapy, and thyroid tumors treated with thyroidectomy, and those with histories of both ICI treatment and thyroid disease (*n* = 2) were excluded (Fig. 1). All patients provided written informed consent. PD-(L)1-Ab treatment was continued until disease progression, death, or development of unacceptable severe adverse events, or if the patient withdrew consent for treatment. This study was approved by the Ethics Committee of Nagoya University Hospital (UMIN000019024).

### Assessments

Thyroid function including serum levels of free T3 (FT3), free T4 (FT4), and thyroid stimulating hormone (TSH) were assessed at baseline and every 6 weeks for 24 weeks after the first administration of PD-(L)1-Ab. After the initial 24 weeks, thyroid function was evaluated at week 36, at week 48, and if clinically needed until the visits stopped. TgAb and TPOAb levels were assessed at baseline and at the onset of thyroid dysfunction. In patients who developed thyrotoxicosis, the TSH receptor antibody (TRAb) level was also measured. Serum levels of FT3, FT4, TSH, TPOAb, TgAb, and TRAb were measured as described previously [24]. Ultrasonography of the thyroid gland was performed in patients who had positive TgAb and/or TPOAb at baseline, as well as those who developed thyroid dysfunction after the initiation of PD-(L)1-Ab treatment. Thyroid dysfunction was defined according to the clinical guidelines of the Japan Endocrine Society [4]. Thyroid function is classified as thyrotoxicosis and hypothyroidism, and thyrotoxicosis is further divided into DT and hyperthyroidism (Graves’ disease). DT was defined as a decreased TSH level, elevated FT3 and/or FT4 levels, and TRAb negativity based on the diagnostic criteria for painless thyroiditis established by the Japan Thyroid Association [26]. Hyperthyroidism was defined as a decreased TSH level, elevated FT3 and/or FT4 levels, increased thyroid uptake of ^99m^Tc pertechnetate on scintigraphy when available, and TRAb positivity based on the diagnostic criteria for Graves’ disease established by the Japan Thyroid Association [26]. Hypothyroidism was defined as an increased TSH level and decreased FT4 level. Isolated hypothyroidism was defined as hypothyroidism without preceding thyrotoxicosis. Subclinical thyroid dysfunction was defined as a TSH level beyond the normal reference range but normal parameters otherwise [27]. The cumulative incidence of thyroid dysfunction was analyzed during the initial year after the first PD-(L)1-Ab administration, since most cases of thyroid dysfunction developed during the initial 6 months, at the latest by 1 year, in our previous studies [9, 10, 24, 25].

### Statistical analysis

Categorical variables are expressed as numbers and percentages and continuous variables as medians (interquartile range). The significance of differences between nominal variables was analyzed using Fisher’s exact test. The significance of differences between continuous variables was tested using the two-sample t test for normally distributed variables or the Mann–Whitney U test for nonnormally distributed variables. Kaplan–Meier curves were used to evaluate the cumulative incidence of thyroid dysfunction, differences in which were determined by log-rank test. Since the PD-(L)1-Ab/TKI group comprised both RCC and gynecologic cancer patients, a subgroup analysis of the incidence of thyroid dysfunction was performed in the RCC patients and gynecologic cancer patients. A multivariate Cox regression model (adjusted for age, sex, ICI type, ATAs at baseline, and TKI usage) was used to identify potential risk factors for the development of thyroid dysfunction. Overall survival (OS), which was determined until death from any cause, was analyzed using the Kaplan–Meier method and compared using a log-rank test; OS is expressed as the median survival time. All statistical tests were two-sided, and significance was defined as a *p* value < 0.05. Statistical analyses were performed using IBM SPSS Statistics 29 (IBM, Armonk, NY).

## Results

### Patient characteristics

A total of 757 patients with malignancies treated with a PD-(L)1-Ab (nivolumab, pembrolizumab, or avelumab, *n* = 734) or PD-(L)1-Ab/TKI (*n* = 23) were enrolled in this study (Fig. 1). The clinical characteristics of the patients treated with PD-(L)1-Ab and PD-(L)1-Ab/TKI are presented in Table 1. There was a significant difference in the tumor type between the PD-(L)1-Ab and PD-(L)1-Ab/TKI groups (Table 1). There was no significant difference in sex, age, or the prevalence of ATA (TgAb and/or TPOAb), TgAb, or TPOAb positivity at baseline between the two groups (Table 1). The PD-(L)1-Abs and TKIs used are shown in Table 1.

**Table 1:** Patient characteristics

	PD-(L)1	PD-(L)1/TKI	*p* value
	(*n* = 734)	(*n* = 23)	
Tumor type			< 0.001
Malignant melanoma	96 (13.1%)	0	
Non-small cell lung carcinoma	165 (22.5%)	0	
Renal cell carcinoma	36 (4.9%)	18 (78.3%)	
Gastric cancer	187 (25.5%)	0	
Head and neck cancer	107 (14.6%)	0	
Urothelial cancer	49 (6.7%)	0	
Mesothelioma	17 (2.3%)	0	
Gynecologic cancer	20 (2.7%)	5 (21.7%)	
Breast cancer	15 (2.0%)	0	
Multiple cancers	8 (1.1%)	0	
Microsatellite instability high tumor	20 (2.7%)	0	
Tumor mutational burden high tumor	3 (0.4%)	0	
Unknown	6 (0.8%)	0	
Other	5 (0.7%)	0	
Sex			
Male	494 (67.3%)	12 (52.2%)	0.175
Female	240 (32.7%)	11 (47.8%)	
Age, years	69 (59–74)	72 (68–75)	0.219
Positive ATAs at baseline (TgAb and/or TPOAb)	123 (16.8%)	4 (17.4%)	1.000
Positive TgAb	63 (8.6%)	4 (17.4%)	0.138
Positive TPOAb	94 (12.8%)	2 (8.7%)	0.756
Treatment			
Pem	307 (41.8%)	0	
Niv	412 (56.1%)	0	
Ave	15 (2.0%)	0	
Pem + axitinib	0	1 (4.3%)	
Pem + lenvatinib	0	8 (34.8%)	
Niv + cabozantinib	0	8 (34.8%)	
Ave + axitinib	0	6 (26.1%)	
Thyroid function during observation period			
Overt thyroid dysfunction	74 (10.1%)	14 (60.9%)	< 0.001
Subclinical thyroid dysfunction	162 (22.1%)	4 (17.4%)	
Euthyroid	498 (67.8%)	5 (21.7%)	

PD-(L)1-Ab, anti-programmed cell death-1 (ligand-1) antibody; TKI, tyrosine kinase inhibitor; ATAs, anti-thyroid antibodies; TgAb, anti-thyroglobulin antibody; TPOAb, anti-thyroid peroxidase antibody; Pem, pembrolizumab; Niv, nivolumab; Ave, avelumab

### The risk of thyroid dysfunction was higher in the PD-(L)1-Ab/TKI group than PD-(L)1-Ab group

**Fig. 2:**
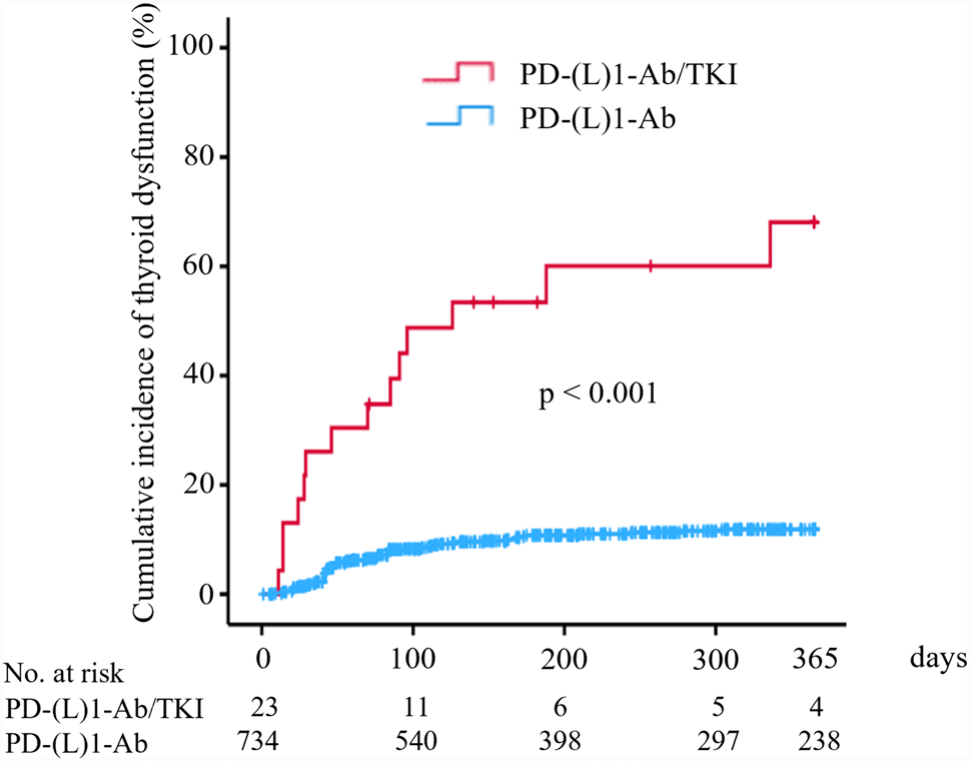
Cumulative incidence of thyroid dysfunction in patients treated with PD-(L)1-Ab/TKI or PD-(L)1-Ab. Kaplan–Meier curves showing patients treated with PD-(L)1-Ab/TKI (red line) or PD-(L)1-Ab (blue line). PD-(L)1-Ab, anti-programmed cell death-1 (ligand-1) antibody; TKI, tyrosine kinase inhibitor

During the observation period, 74 (10.1%) and 14 (60.9%) patients developed thyroid dysfunction, and 162 (22.1%) and 4 (17.4%) patients developed subclinical thyroid dysfunction after the initiation of PD-(L)1-Ab and PD-(L)1-Ab/TKI treatment, respectively (Table 1). The cumulative incidence of thyroid dysfunction was significantly higher in the PD-(L)1-Ab/TKI group than PD-(L)1-Ab group (log-rank test, *p* < 0.001) (Fig. 2). The clinical characteristics of the thyroid dysfunction between the PD-(L)1-Ab and PD-(L)1-Ab/TKI groups are presented in Table 2.　There was a significant difference in tumor type between the PD-(L)1-Ab and PD-(L)1-Ab/TKI groups. Regarding the type of thyroid dysfunction, the incidence of isolated hypothyroidism was significantly higher in the PD-(L)1-Ab/TKI group than PD-(L)1-Ab group [10/14 (71.4%) vs. 29/74 (39.2%) patients, *p* < 0.05]. There was no significant difference in age, sex, incidence of levothyroxine treatment, or the median number of days from the first administration to the diagnosis of thyroid dysfunction between the two groups, respectively (Table 2). In the multivariate Cox regression analysis, ATAs at baseline [odds ratio (OR) 6.970, 95% confidence interval (CI) 4.500–10.796, *p* < 0.001] and TKI usage (OR 7.578, 95% CI 4.048–14.187, *p* < 0.001) were significantly associated with the development of thyroid dysfunction (Supplementary Table 1).

**Table 2:** Clinical characteristics of the patients who developed thyroid dysfunction induced by PD-(L)1-Ab monotherapy or PD-(L)1-Ab plus TKI combination therapy

	PD-(L)1	PD-(L)/TKI	*p* value
	*n* = 74	*n* = 14	
Tumor type			
Malignant melanoma	17 (23.0%)	0	< 0.001
Non-small cell lung carcinoma	12 (16.2%)	0	
Renal cell carcinoma	6 (8.1%)	10 (71.4%)	
Gastric cancer	11 (14.9%)	0	
Head and neck cancer	15 (20.3%)	0	
Urothelial cancer	3 (4.1%)	0	
Mesothelioma	1 (1.4%)	0	
Gynecologic cancer	4 (5.4%)	4 (28.6%)	
Breast cancer	4 (5.4%)	0	
Microsatellite instability high tumor	1 (1.4%)	0	
Age, years	69 (58–76)	70 (66–73)	0.501
Sex			
Male	39 (52.7%)	6 (42.9%)	0.568
Female	35 (47.3%)	8 (57.1%)	
Type of thyroid dysfunction			
Destructive thyroiditis	45 (60.8%)	4 (28.6%)	0.039
Isolated Hypothyroidism	29 (39.2%)	10 (71.4%)	
LT4 treatment required	59 (79.7%)	13 (92.9%)	0.450
Days to diagnosis from the first administration			
Thyroid dysfunction	48 (41–96)	58 (22–104)	0.711
Destructive thyroiditis	42 (30–53)	29 (25–259)	0.609
Isolated Hypothyroidism	110 (73–168)	78 (14–104)	0.112

PD-(L)1-Ab, anti-programmed cell death-1 (ligand-1) antibody; TKI, tyrosine kinase inhibitor; LT4, levothyroxine

### High incidences of DT and isolated hypothyroidism induced by PD-1-Ab/TKI treatment

**Fig. 3:**
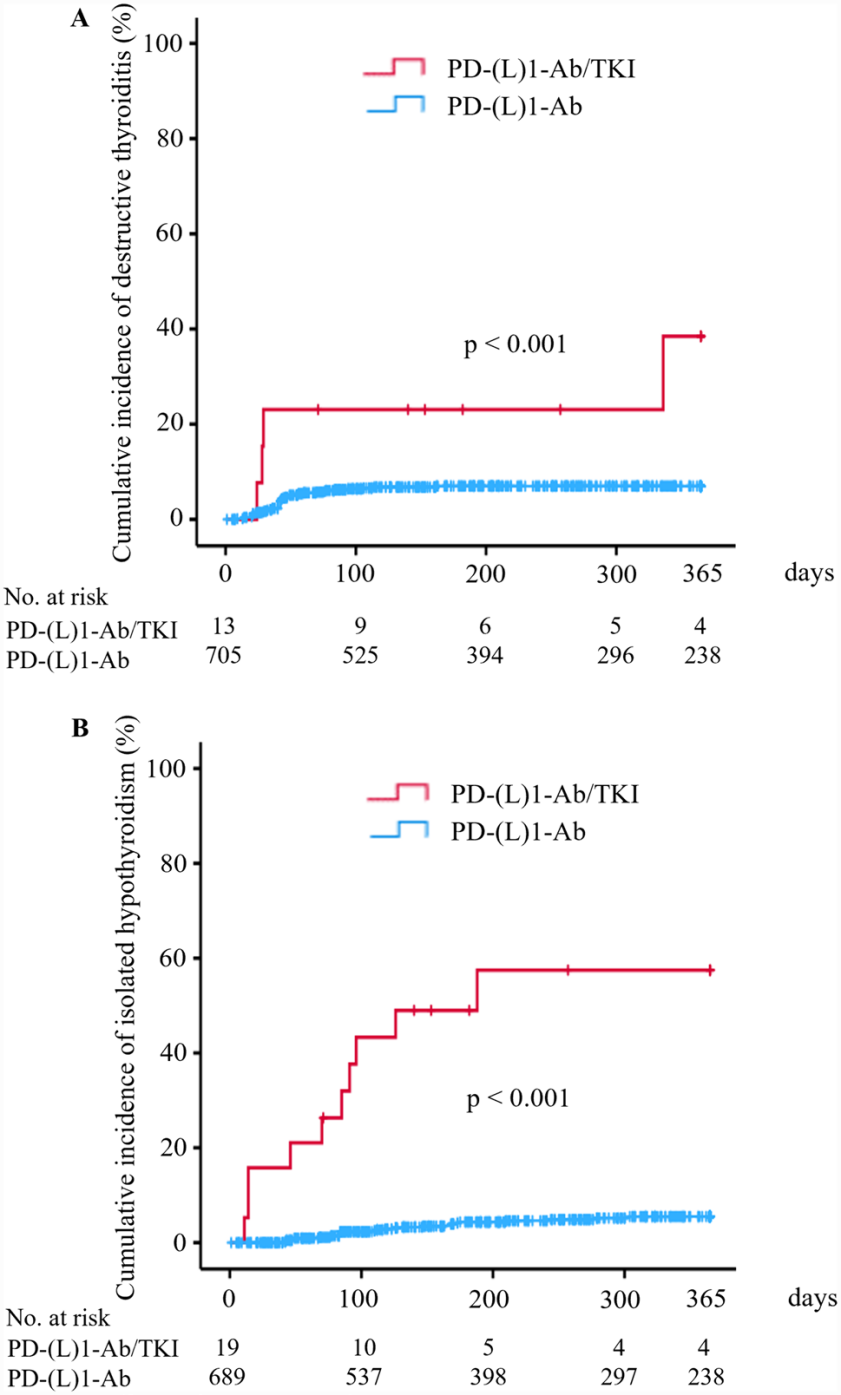
Cumulative incidences of DT and isolated hypothyroidism in patients treated with PD-(L)1-Ab/TKI or PD-(L)1-Ab. Kaplan–Meier curves showing patients who developed DT (**A**) and patients who developed isolated hypothyroidism (**B**) after PD-(L)1-Ab/TKI (red line) or PD-(L)1-Ab (blue line) treatment, respectively. PD-(L)1-Ab, anti-programmed cell death-1 (ligand-1) antibody; TKI, tyrosine kinase inhibitor

During the observation period, 45 (6.1%) and 4 (17.4%) patients developed thyrotoxicosis after initiation of the PD-(L)1-Ab and PD-(L)1-Ab/TKI treatments, respectively, all of whom were diagnosed with DT based on TRAb negativity (Table 2). The cumulative incidence of DT was significantly higher in the PD-(L)1-Ab/TKI group than PD-(L)1-Ab group (log-rank test, *p* < 0.001, Fig. 3A). There was no significant difference in the median number of days from the first administration to the diagnosis of DT between the two groups (Table 2). Furthermore, 29 (4.0%) and 10 (43.5%) patients developed isolated hypothyroidism after initiation of the PD-(L)1-Ab and PD-(L)1-Ab/TKI treatments, respectively. The cumulative incidence of isolated hypothyroidism was significantly higher in the PD-(L)1-Ab/TKI group than PD-(L)1-Ab group (log-rank test, *p* < 0.001, Fig. 3B). There was no significant difference in the median number of days from the first administration to the diagnosis of isolated hypothyroidism between the two groups (Table 2). In a subgroup analysis among patients with RCC and patients with gynecologic cancer, the cumulative incidences of thyroid dysfunction [14/23 (60.9%) vs. 10/56 (17.9%) patients, *p* < 0.001] and isolated hypothyroidism [10/23 (43.5%) vs. 3/56 (5.4%) patients, *p* < 0.001] were significantly higher in the PD-(L)1-Ab/TKI group than the PD-(L)1-Ab group, respectively (Supplementary Fig. 1A, C). Although a similar tendency in the incidence of DT was observed, there was no significant difference between the two groups [4/23 (17.4%) vs. 7/56 (12.5%) patients, *p* = 0.105] (Supplementary Fig. 1B).

### Association of ATA positivity at baseline with the incidence of thyroid dysfunction

**Fig. 4:**
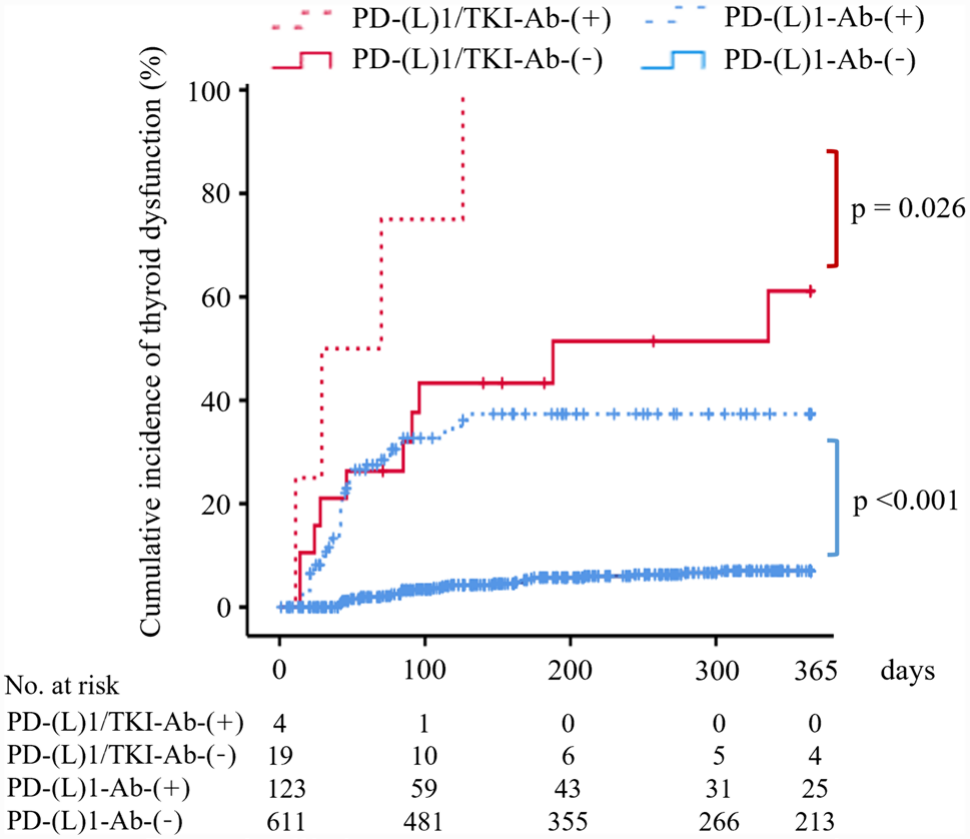
Cumulative incidence of thyroid dysfunction in patients treated with PD-(L)1-Ab/TKI and PD-(L)1-Ab. Kaplan–Meier curves showing patients treated with PD-(L)1-Ab/TKI (red line) or PD-(L)1-Ab (blue line). Dashed and solid lines indicate the patients positive and negative for anti-thyroid antibodies at baseline, respectively. PD-(L)1-Ab, anti-programmed cell death-1 (ligand-1) antibody; TKI, tyrosine kinase inhibitor; Ab, anti-thyroid antibody

At baseline, of the patients treated with PD-(L)1-Ab or PD-(L)1-Ab/TKI, ATA (TgAb and/or TPOAb) positivity was observed in 123 (16.8%) [PD-(L)1-Ab-(+) group] and 4 (17.4%) [PD-(L)1/TKI-Ab-(+) group] patients, respectively (Table 1); the remaining 611 and 19 ATA-negative patients were designated as the PD-(L)1-Ab-(−) and PD-(L)1/TKI-Ab-(−) groups, respectively. During the observation period after ICI initiation, the cumulative incidence of thyroid dysfunction was significantly different among the four groups (log-rank test, *p* < 0.001, Fig. 4). In particular, the incidence was higher in the PD-(L)1-Ab-(+) group than PD-(L)1-Ab-(−) group [41/123 (33.3%) vs. 33/611 (5.4%) patients, log-rank test, *p* < 0.001] and in the PD-(L)1/TKI-Ab-(+) group than PD-(L)1/TKI-Ab-(－) group [4/4 (100%) vs. 10/19 (52.6%) patients, log-rank test, *p* = 0.026] (Fig. 4). Among the patients positive for ATAs at baseline, the cumulative incidence of thyroid dysfunction was significantly higher in the PD-(L)1/TKI-Ab-(+) group than PD-(L)1-Ab-(+) group (100% vs. 33.3% of patients, log-rank test, *p* = 0.002) (Fig. 4). Notably, among the patients negative for ATAs at baseline, the cumulative incidence of thyroid dysfunction was significantly higher in the PD-(L)1/TKI-Ab-(−) group than PD-(L)1-Ab-(−) group (52.6% vs. 5.4% of patients, log-rank test, *p* < 0.001) (Fig. 4). The risk of thyroid dysfunction was not significantly different between the PD-(L)1/TKI-Ab-(−) and PD-(L)1-Ab-(+) groups (*p* = 0.244).

### OS was not different between the PD-(L)1-Ab and PD-(L)1-Ab/TKI groups among the RCC or gynecologic *cancer* patients.

Next, we compared OS between patients in the PD-(L)1-Ab and PD-(L)1-Ab/TKI groups. Since the PD-(L)1-Ab/TKI group comprised both RCC and gynecologic cancer patients, OS was analyzed in these patients. There was no significant difference in OS between the PD-(L)1-Ab and PD-(L)1-Ab/TKI groups among the patients with RCC (median: 1237 days vs. not reached, *p* = 0.099) or those with gynecologic cancer (median: not reached vs. 650 days, *p* = 0.883), respectively.

## Discussion

This prospective study, in which thyroid hormone levels were measured at regular intervals, demonstrated that combined use of a TKI increased the incidence of thyroid dysfunction induced by PD-(L)1-Ab treatment, and that ATA positivity at baseline was associated with an increased risk of developing thyroid dysfunction after PD-(L)1-Ab/TKI treatment. The association between TKI usage and the development of thyroid dysfunction was confirmed by multivariate Cox regression analysis. Furthermore, the increased risk of thyroid dysfunction after PD-(L)1-Ab/TKI treatment was not due to the extended observation period resulting from longer survival times.

The thyroid dysfunction induced by TKIs reported so far has been primarily hypothyroidism [28], which could explain the higher prevalence of isolated hypothyroidism induced by PD-(L)1-Ab/TKI in this study (Table 2). On the other hand, while the incidence of DT has been rarely evaluated in clinical trials of TKI treatment, a prospective study including 69 patients with RCC showed that transient thyrotoxicosis preceded TKI-induced hypothyroidism in 23.9% [29] of patients, suggesting that development of TKI-induced DT, which generally was not evaluated as a primary outcome, might have been overlooked in most previous clinical trials. Our prospective study showing that the DT incidence was higher after PD-(L)1-Ab/TKI than PD-(L)1-Ab treatment further supports this possibility. Since the subgroup analysis showed no significant difference in the incidence of DT among the RCC or gynecologic cancer patients, further analysis using a larger sample size is required.

Several studies have shown that CD4 + and CD8 + T cells infiltrate the thyroid gland in patients who develop PD-1-Ab-induced DT, suggesting that PD-1-Abs activate autoreactive T cells against the thyroid glands [30–32]. In a mouse model, we also reported that PD-1-Ab injections induced activation of cytotoxic CD4 + memory T cells, directly damaging thyroid cells [33]. On the other hand, TKIs can also increase autoimmunity via mechanisms such as enhancing T cell function and decreasing the number of immunosuppressive cells including regulatory T cells [34, 35]. Thus, the combined use of a PD-(L)1-Ab and TKI might cooperatively enhance the activation of T cells targeting the thyroid gland. In addition, there are reports of TKIs reducing thyroid vascular density and fenestrations via blockade of VEGF receptor signaling [13, 36, 37], inhibiting iodine uptake [38] as well as TPO activity [39], and enhancing type 3 deiodinase activity, all of which could have contributed to thyroidal dysfunction induced by PD-(L)1-Ab/TKI. Therefore, thyroid dysfunction may be more severe with PD-(L)1-Ab/TKI treatment than with PD-(L)1-Ab treatment. This hypothesis was supported by the higher incidence of overt thyroid dysfunction in the PD-(L)1-Ab/TKI group than the PD-(L)1-Ab group.

We previously reported that ATA positivity at baseline is a risk factor for thyroid dysfunction induced by PD-(L)1-Ab treatment [9, 10, 24, 25, 40–43], which was also confirmed by multivariate Cox regression analysis in this study. The present study also showed that ATA positivity at baseline is associated with the increased incidence of PD-(L)1-Ab/TKI-induced thyroid dysfunction; all four cases positive for ATAs at baseline developed thyroid dysfunction. It is also of note that the incidence of thyroid dysfunction was high even among the patients negative for ATAs at baseline in the PD-(L)1-Ab/TKI group, which was almost equivalent to that among patients positive for ATAs at baseline in the PD-(L)1-Ab group.

This study has several limitations. First, the sample size of the PD-(L)1-Ab/TKI group was relatively small because only a limited number of malignancies could be treated with PD-(L)1-Ab/TKI during the study period. Second, since the TKIs (lenvatinib, cabozantinib, and axitinib) were analyzed together as a single group, it was not possible to detect differences in the incidence of thyroid dysfunction among the three TKIs employed. Further investigation using a larger sample size for each treatment is needed. Third, there was a significant difference in the tumor types between the PD-(L)1-Ab and PD-(L)1-Ab/TKI groups. Further studies with larger sample sizes are needed to clarify the difference, if any, among the drugs and cancer types. Fourth, a comparison of the clinical characteristics of thyroid dysfunction in patients treated with PD-(L)1-Ab/TKI versus TKI monotherapy was not possible because our prospective study included only patients treated with ICIs.

In conclusion, TKIs increased the incidence of thyroid dysfunction induced by PD-(L)1-Ab, the risk of which is even higher in patients positive than in those negative for ATAs at baseline.

### Supplementary Information

Below is the link to the electronic supplementary material.

Supplementary file1 (PDF 201 KB)

## Data Availability

No datasets were generated or analyzed during the current study.
