# Supplementary material for: Combined use of tyrosine kinase inhibitors with PD-(L)1 blockade increased the risk of thyroid dysfunction in PD-(L)1 blockade: a prospective study
Source: Cancer Immunol Immunother. 2024 Jun 4;73(8):146. doi: 10.1007/s00262-024-03733-2 (PMC11150211; doi:10.1007/s00262-024-03733-2)
Supplement: Supplementary file 1 — Supplementary file1 (PDF 201 KB) [file 262_2024_3733_MOESM1_ESM.pdf]

Supplementary Table 1. Risk factors for the development of thyroid dysfunction

Covariate		Multivariate analysis		
		Odds ratio	(95% CI)	p value
Age	≥69 vs. <69 years	0.851	(0.556–1.301)	0.456
Sex	Female vs. male	1.512	(0.970–2.359)	0.068
ICI type	PD-1 Ab vs. PD-L1 Ab	0.688	(0.226–2.094)	0.510
ATAs at baseline	Positive vs. negative	6.970	(4.500–10.796)	<0.001
TKI usage	Positive vs. negative	7.578	(4.048–14.187)	<0.001

Abbreviations: CI, confidence interval; ICI, immune checkpoint inhibitor; PD-1 Ab, anti-programmed cell death-1 antibody; PD-L1 Ab, anti-programmed cell death-1 ligand 1 antibody; ATAs, anti-thyroid antibodies (anti-thyroglobulin antibody and/or anti-thyroid peroxidase antibody); TKI, tyrosine kinase inhibitors.

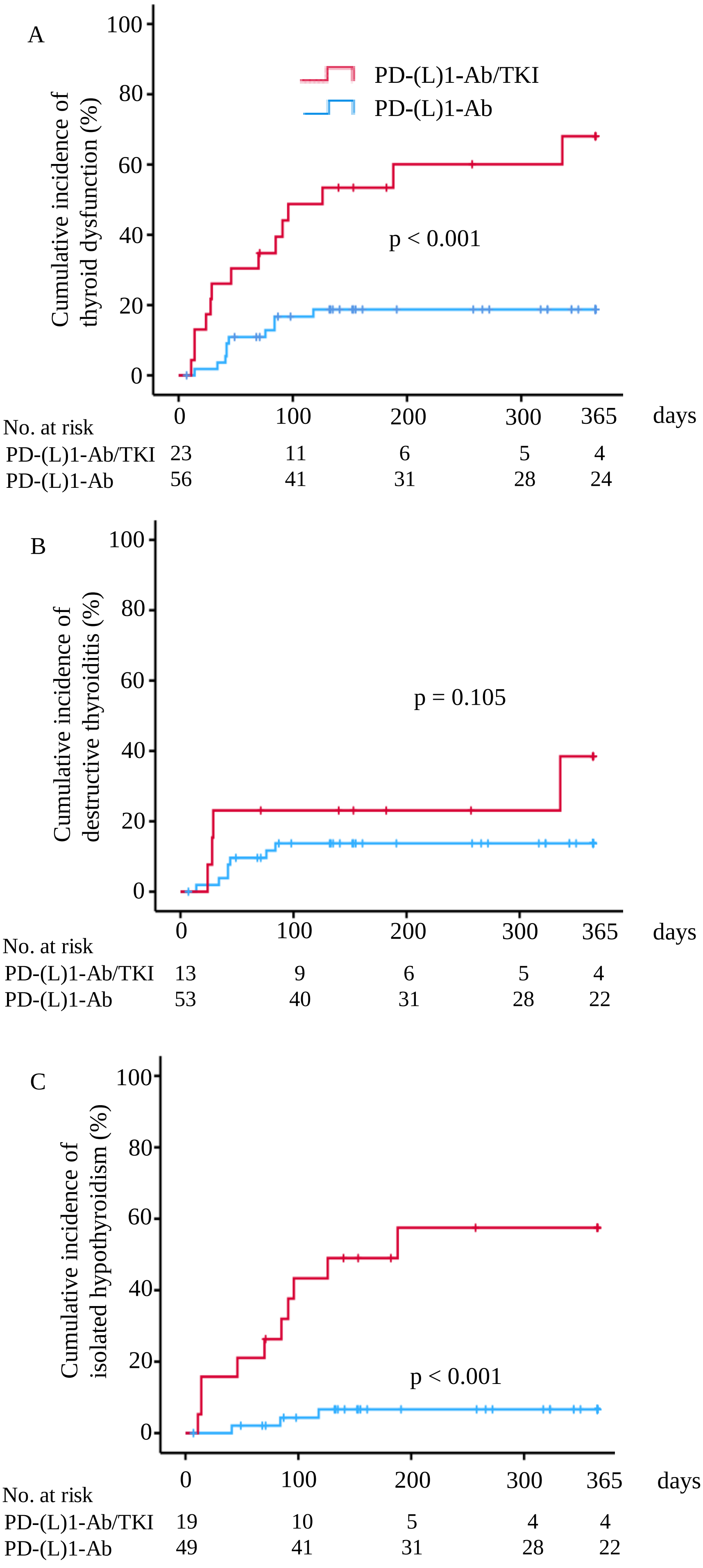

Supplementary Figure 1

Supplementary Figure 1. Cumulative incidence of thyroid dysfunction in patients with renal cell carcinoma or gynecologic cancer

Kaplan–Meier curves showing patients who developed thyroid dysfunction (A), DT (B), or isolated hypothyroidism (C) after PD-(L)1-Ab/TKI (red line) or PD-(L)1-Ab (blue line) treatment among the patients with RCC and those with gynecologic cancer. Abbreviations: PD-(L)1-Ab, anti-programmed cell death-1 (ligand-1) antibody; TKI, tyrosine kinase inhibitor.
